# Cytoreductive Surgery for Heavily Pre-Treated, Platinum-Resistant Epithelial Ovarian Carcinoma: A Two-Center Retrospective Experience

**DOI:** 10.3390/cancers12082239

**Published:** 2020-08-10

**Authors:** Valentina Tuninetti, Marilena Di Napoli, Eleonora Ghisoni, Furio Maggiorotto, Manuela Robella, Giulia Scotto, Gaia Giannone, Margherita Turinetto, Dimitris Siatis, Riccardo Ponzone, Marco Vaira, Michele De Simone, Cono Scaffa, Sandro Pignata, Stefano Greggi, Massimo Di Maio, Giorgio Valabrega

**Affiliations:** 1 Candiolo Cancer Institute, FPO-IRCCS-Candiolo, 10060 Torino, Italy; valentina.tuninetti@ircc.it (V.T.); furio.maggiorotto@ircc.it (F.M.); manuela.robella@ircc.it (M.R.); giulia.scotto@ircc.it (G.S.); gaia.giannone@ircc.it (G.G.); margherita.turinetto@ircc.it (M.T.); dimitris.siatis@ircc.it (D.S.); riccardo.ponzone@ircc.it (R.P.); marco.vaira@ircc.it (M.V.); michele.desimone@ircc.it (M.D.S.); giorgio.valabrega@ircc.it (G.V.); 2 Department of Oncology, University of Torino, 10043 Torino, Italy; massimo.dimaio@unito.it; 3 Department of Urology and Gynecology, Istituto Nazionale Tumori IRCCS Fondazione G. Pascale, 80131 Napoli, Italy; m.dinapoli@istitutotumori.na.it (M.D.N.); s.pignata@istitutotumori.na.it (S.P.); 4 Department of Gynecologic Oncology Surgery, Istituto Nazionale Tumori IRCSS Fondazione G. Pascale, 80131 Napoli, Italy; c.scaffa@istitutotumori.na.it (C.S.); s.greggi@istitutotumori.na.it (S.G.); 5 Medical Oncology, Ordine Mauriziano Hospital, 10028 Torino, Italy

**Keywords:** cytoreductive surgery, platinum resistance, epithelial ovarian carcinoma

## Abstract

Few retrospective studies have shown a benefit in selected patients affected by heavily pre-treated, platinum-resistant ovarian carcinomas (PROCs) who have undergone cytoreduction at relapse. However, the role of tertiary and quaternary cytoreductive surgery is not fully defined. Our aim was to evaluate survival and surgical morbidity and mortality after maximal cytoreduction in this setting. We evaluated all consecutive patients undergoing cytoreduction for platinum-resistance over an 8-year period (2010–2018) in two different centers. Fifty patients (median age 52.5 years, range 34–75) were included; the median number of previous chemotherapy lines was three (range 1–7) and the median number of previous surgeries was one (range 1–4). Completeness of cytoreduction (CC = 0) was achieved in 22 patients (44%). Rates of major operative morbidity and 30-day mortality were 38% and 8%, respectively. Median follow-up was 35 months. The absence of tumor residual (CC = 0) was associated with a significantly better overall survival (OS) compared to the CC > 0 subgroup (median OS 32.9 months (95% CI 21.6–44.2) vs. 4.8 months (95% CI n.a.–9.8), hazard ratio (HR) 4.21 (95% CI 2.07–8.60), *p* < 0.001). Optimal cytoreduction is feasible and associated with promising OS in selected, heavily pre-treated PROCs. Further prospective studies are required to better define the role of surgery in platinum-resistant disease.

## 1. Introduction

Ovarian cancer (OC) is the leading cause of death from gynecological malignances with an estimated 21,750 new cases and 13,940 deaths in 2020 in the USA [1].

Optimal surgical cytoreduction preceded and/or followed by platinum-based chemotherapy is currently the standard of care for advanced OC [2] and the absence of residual disease (Completeness of cytoreduction [CC = 0]) at primary surgery has yet demonstrated to be the most relevant prognostic factor for overall survival (OS) [2,3,4]. However, despite multimodal treatment, approximately 70% of patients with advanced stage disease (FIGO stage III or IV) relapse within 2 years and ultimately develop platinum-resistant disease [5].

Several authors have tried to define the role of surgery in the relapsed setting of the disease.

In the context of platinum-sensitive disease, two phase III randomized controlled trials, the DESKTOP III [6,7] and the Gynecologic Oncology Group (GOG) 213 studies [8,9], analyzed the therapeutic impact of secondary cytoreductive surgery (SCS). The final results of the DESKTOP III trial showed that SCS in patients with a positive Arbeitsgemeinschaft Gynäkologische Onkologie (AGO) score (defined as complete resection at first surgery, good performance status (PS) and the absence of ascites) resulted in a median progression-free survival (PFS)benefit of 5.6 months (14 versus 19.6 months, *p* < 0.001) and a benefit in the median time to the start of subsequent chemotherapy of 7.1 months (13.9 versus 21 months, *p* < 0.001). The benefit in terms of PFS was observed only in patients with optimal cytoreduction [6]. Even more relevant is the benefit in terms of OS in the surgery arm (median 61.9 months for complete resection versus 46 months in the non-surgical arm, versus 28.8 months in the incomplete debulking subgroup). Mortality rate and grade 3 and 4 adverse events (AEs) were similar in both arms [7].

Similarly, the significant improvement of PFS with the addition of SCS have also been reported in the GOG 213 trial but again only in patients with complete resection. By contrast, OS did not show a statistically significant improvement with the addition of surgery [9].

The role of tertiary and quaternary cytoreductive surgery in patients with second and third recurrences is not fully defined, although several retrospective studies showed a benefit in highly selected patients who underwent complete cytoreduction at relapse [10,11,12,13,14,15,16].

Even less data, from a limited numbers of patients, are available on the role of surgery in platinum-resistant ovarian cancer (PROC), defined as the last platinum-free interval (PFI) being < 6 months. Petrillo et al. [17] analyzed the role of surgery in PROCs and demonstrated that, following complete resection, a possible survival benefit may be achieved. Moreover, Musella et al. [18] reported longer OS in 18 patients treated with SCS for platinum resistance (PR) compared with 18 patients not suitable for surgery (67 months vs. 24 months, respectively); El Halabi et al. [19] reported prolonged PFS in 53 patients treated with cytoreductive surgery and heated intraperitoneal chemotherapy (HIPEC) for PR (median PFS 10.3 months post HIPEC versus 4.4 months for the penultimate treatment prior to HIPEC) (*p* < 0.001).

In this study, we aimed to describe the characteristics and outcomes of heavily pre-treated PROC patients who underwent surgery in two different institutions. We report the surgical procedures and outcomes including morbidity and mortality.

## 2. Materials and Methods

We retrospectively retrieved the medical records of 897 patients with a histologically confirmed diagnosis of epithelial ovarian cancer (EOC) operated on between June 2010 and December 2018 at Candiolo Cancer Institute, FPO-IRCCS and at the National Cancer Institute G. Pascale Foundation, Napoli. Patients who underwent surgery for PR disease were selected. Eligibility for surgery was based on the probability of optimal cytoreduction, after discussion within the gynecological tumor board and the patients were deeply informed about the risks and possible alternative treatments. We recorded the following data: (1) age at time of diagnosis and at surgery for PR; (2) morphological features of EOC, including tumor histotype, grade (according to the World Health Organization Classification of Tumors of Female Reproductive Organs, 4th Edition) and stage (according to the International Federation of Gynecology and Obstetrics (FIGO)); (3) type of surgery at diagnosis (primary debulking surgery (PDS) versus interval debulking surgery (IDS)); (4) residual disease according to the CC system at first surgery and for PR [20]; (5) peritoneal cancer index (PCI) according to Sugarbaker [21,22] at first surgery and for PR; (6) PFI to last platinum-based therapy; (7) number of previous lines of chemotherapy and previous surgical procedures before surgery for PROC; (8) surgical procedures and major complications; (9) date of death or last follow-up (FU).

### 2.1. Institutional Review Board (IRB) Approval

In Italy, the National Regulation established that retrospective studies require a notification to the local ethical committee with the tacit consent formula. Due to the retrospective nature of this study, the local institutional review board (FPO-IRCCS ethical committee) waived the requirement for additional approval. We therefore notified the FPO-IRCCS ethical committee about the conduction of the study in October 2018. The patients’ informed consent was always given before surgery and sample collection and documentation.

### 2.2. Statistics

All analyses were performed using the SPSS statistical software program, version 26.0 (IBM SPSS Inc., Chicago, IL, USA). Estimates of survival were calculated by the Kaplan–Meier method and compared using the logrank test. The relative importance of variables as predictors of OS was analyzed with the multivariate Cox proportional hazard regression. A *p* < 0.05 was considered statistically significant. The FU time was calculated starting on the day of surgery for PR, based on the reverse Kaplan–Meier method [23].

## 3. Results

A total of 50 patients who underwent surgery for platinum-resistant disease were identified.

At diagnosis, the majority of patients had serous (90%), advanced (80% stage FIGO III or IV) OC. Thirty-seven patients (74%) had PDS at diagnosis, while 11 patients (22%) received neoadjuvant chemotherapy (NACT) followed by IDS. The absence of residual disease (CC = 0) was obtained in 16 patients (32%) and all patients had a first line platinum-based chemotherapy. Breast cancer (BRCA) status was known in 15 (30%) patients (10% showed a BRCA 1 mutation, 2% BRCA 2 mutation, 6% a variant of unknown significance (VUS) and 10% had wild-type BRCA). The median PFI at first recurrence was 7.5 months (interquartile range 3–13.75). Thirty (60%) were platinum-sensitive at first relapse, while 20 (40%) patients had primary platinum-resistant disease. At first recurrence, secondary surgery was performed in 18 patients (36%). Complete baseline patient clinical data are reported in Table 1.

Median time from primary surgery to surgery for platinum-resistant disease was 29.0 months (interquartile range 12.9–55.4 months) and 39 patients (78%) underwent surgery within 5 years. Thirty-two (64%) received chemotherapy before surgery for platinum-resistant recurrence. The median number of previous chemotherapy lines before surgery for PR was three (range 1–7) and the median number of previous surgical procedures was one (range 1–4). The median age at surgery for platinum-resistant relapse was 56.5 years (range 35.6–78.4). The majority of patients (96%) showed a diffuse pattern of recurrence involving multiple anatomic sites. Only two patients presented an isolated recurrence. The most common sites of recurrence were peritoneum (31/50, 60%), followed by lymph nodes (22/50, 44%), pelvis and liver (8/50, 16%). Detailed tumor dissemination patterns are described in Table 2.

The primary objective of all the operations was optimal cytoreduction. Of note, seven patients (14%) were operated on due to bowel obstruction, but even in these cases an extended resection was performed to remove a large part of the tumor together with a definitive stoma.

In all patients, a laparotomic and multi-visceral surgical approach was used. Out of 39 patients with information available, the median duration of surgery was 170 min (range 30–660 min).

Twenty-three patients (54%) had PCI < 16 at surgery (range 0–36). Twenty-seven patients (54%) had no intraoperative ascites and an optimal residual tumor (CC-0) was achieved in 27 patients out of 50 (54%). The most frequent surgical procedures performed were lymph node dissection (44%), intestinal resection (44%), peritonectomy (36%), omentectomy (32%) and splenectomy (16%). Partial liver resection and cholecystectomy were performed in 10% of the cases.

The rates of major operative morbidity and 30-day mortality were 38% and 8%, respectively. Pneumothorax (10%), acute respiratory failure (4%), lymphocele (4%), acute renal insufficiency (4%) and infection (4%) were the most frequent complications. Details are listed in Table 2.

Twenty patients (40%) received post-operative chemotherapy (i.e., cyclophosphamide, pegylated liposomal doxorubicin) while 20 (40%) continued with FU (in 10 cases, the information is not available).

**Figure 1:**
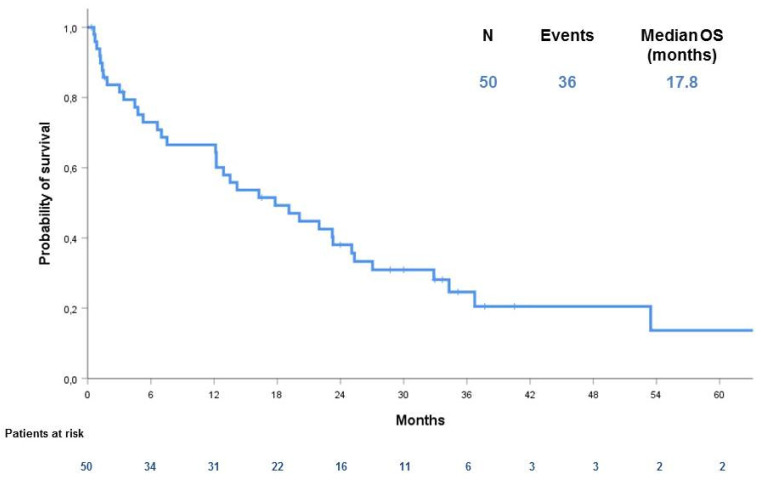
Overall survival (OS) in our case series.

At a median FU of 35 months, 32 patients (64%) had died due to disease progression (PD). The median OS was 17.8 months (CI 95% 9.2–26.4) in the entire series (Figure 1).

**Figure 2:**
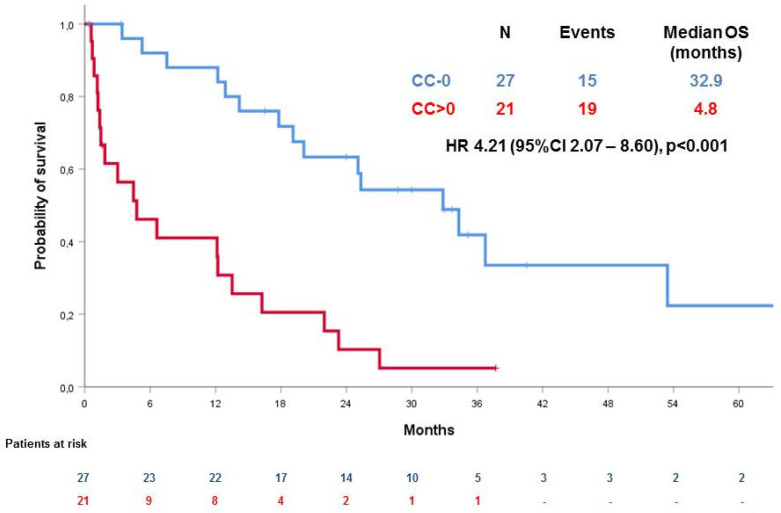
Overall survival (OS) according to residual disease (CC-0 versus not).

The absence of tumor residue (CC = 0) was associated with a significantly better OS in comparison with the presence of tumor residue (CC > 0), (median OS 32.9 months (95% CI 21.6–44,2) vs. 4.8 months (95% CI n.a.–9.8), hazard ratio (HR) 4.21 (95% CI 2.07–8.60), *p* < 0.001) (Figure 2).

**Figure 3:**
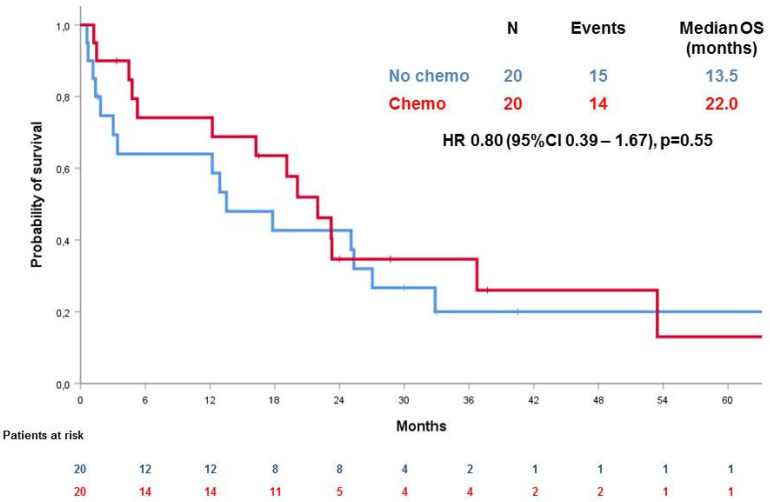
Overall survival (OS) according to post-operatory chemotherapy.

Interestingly, as shown in Figure 3, OS was not significantly different for women receiving or not receiving post-operatory chemotherapy (CT) (*p* = 0.55, HR 0.80 (95% CI 0.39–1.67)), both in the CC-0 group and CC > 0 group (HR 1.04, 95% CI 0.33–3.27, *p* = 0.95 in the CC = 0 subgroup and HR 0.53, 95% CI 0.20–1.47, *p* = 0.22 in the CC > 0 subgroup).

At univariate analysis, ascites was significantly prognostic in terms of OS (median OS 6.6 months for patients with ascites vs. 23.3 months for patients without ascites, *p* = 0.008). No other factors were identified to be prognostic in terms of OS: in particular, PCI (median OS 6.6 months for PCI ≥ 16 vs. 20.1 months for PCI < 16, *p* = 0.14); upper abdomen procedures (median OS 16.3 months for those who did not receive upper abdomen procedures vs. 25.1 months for those who did, *p* = 0.13), age (median OS 17.8 months for patients younger than 65 vs. 13.5 months for patients older than 65, *p* = 0.89), grading (median OS 14.2 months for G < 3 vs. 19.1 months for G3, *p* = 0.61), FIGO stage (median OS 12.1 months for lower stages vs. 20.1 months for stage IIIC-IV, *p* = 0.86).

In the multivariable analysis, including all the above-listed factors, only the absence or presence of tumor residue after surgery was significantly associated with OS (Table 3).

## 4. Discussion

It is well established that cytoreductive surgery plays an increasingly relevant role in platinum-sensitive recurrent ovarian cancer (ROC), with most studies reporting complete resection as an independent prognostic factor for improved survival [24,25,26,27,28,29]. The DESKTOP I and II trials [30,31] identified and validated the AGO score for the prediction of complete secondary cytoreduction in platinum-sensitive ROC.

Nevertheless, few data are available in the setting of PROC and no predictive scores for complete cytoreduction have been validated until now.

This is the largest case series of women undergoing cytoreductive surgery for PROC in two different institutions. In our study, we show that the possibility of performing optimal cytoreductive surgery at the time of recurrence is feasible and associated with the longer survival of these patients. In fact, women with no residual tumor (CC = 0) show a significantly longer survival that those undergoing suboptimal surgery (median OS 32.9 months (95% CI 21.6–44,2) vs. 4.8 months (95% CI n.a.–9.8), hazard ratio (HR) 4.21 (95% CI 2.07–8.60), *p* < 0.001). Moreover, we show that perioperative chemotherapy does not affect the survival of patients in the CC = 0 or in the CC > 0 subgroups (HR 1.04, 95% CI 0.33–3.27, *p* = 0.95 in the CC = 0 subgroup and HR 0.53, 95% CI 0.20–1.47, *p* = 0.22 in the CC > 0 subgroup).

Our results are consistent with those already reported in other single institution experiences, where maximal surgical effort, with minimal/absent residual disease, produced a significant survival benefit. [11,30,32,33,34,35]. However, it should be pointed out that, in contrast with most of the other selected series, our group of platinum-resistant patients is extremely unfavorable, with high tumor load (40% PCI ≥ 16) and highly chemo-resistant disease (median previous lines = 3).

Indeed, it is important to highlight that most patients underwent primary surgery in other institutions with low patient volume, with a very high rate of incomplete resection at primary surgery (50%), thus suggesting an even worse prognosis related to persistent disease rather than recurrent disease.

Despite the extensive tumor dissemination in the upper abdomen and peritoneum, optimal cytoreduction with no residual disease was achieved in 54% of the patients at the cost of reasonable peri-operative morbidity and mortality, although this was more frequent than in reports by Fotopoulou et al. [10] and Musella et al. [18].

Moreover, these percentages appear even more reasonable if we consider the amount of prior cytotoxic therapies and surgeries in our series.

Most importantly, post-surgical tumor residue is also highly prognostic in this highly unfavorable platinum-resistant setting.

Our findings underline once more the crucial value of aggressive high-quality surgery performed in high-volume, specialized centers with appropriate facilities and experience [36].

Despite the encouraging results, several limitations characterize our study. Clearly, the retrospective nature of our analysis affects the reliability of our conclusions resulting in the dimness of indications for surgery and of the criteria for patient selection. However, the similar distribution of baseline clinical and pathological features between the optimally cytoreduced and not optimally cytoreduced groups (Table 1) supports our results. Additionally, we did not evaluate the impact on quality of life (QoL) after cytoreductive surgery; therefore, even if few complications were documented, it cannot be excluded that the longer survival with surgery might have been achieved with an impairment of QoL.

In conclusion, we observed that the complete resection of platinum-resistant disease is associated with longer patient survival, while we did not find a significant difference in the outcome according to the administration of post-operatory chemotherapy. Even though we acknowledge that our analysis includes only two centers, it is non-randomized and it has a limited number of patients, our data support the conduction of prospective trials to better define the role of maximal therapeutic effort in this unfavorable population without any other valuable therapeutic option.

Further studies on higher numbers of patients are required in order to identify patients who could most highly benefit from this procedure.

## 5. Conclusions

Optimal cytoreduction, in our series, was associated with better OS in selected, heavily pre-treated PROC, while peri-operative systemic CT showed no significant impact on OS. However, our retrospective analysis does not allow us to rule out the potential impact of selection bias. Further prospective studies are required to better define the role of surgery for platinum-resistant disease.

## Figures and Tables

**Table 1:** Patient tumor-related and surgical characteristics.

Variables	Patients *N* = 50 (%)
**Median age at first surgery (years, range)**	52.6 (31.9–75.1)
**FIGO stage at primary diagnosis**	
I	2 (4%)
II	3 (6%)
III A	2 (4%)
III B	3 (6%)
III C.	31 (62%)
IV	4 (8%)
Not available	5 (10%)
**Histology**	
Serous	45 (90%)
Endometrioid	2 (4%)
Clear cell	2 (4%)
Not available	1 (2%)
**Grading**	
G1	1 (2%)
G2	4 (8%)
G3	40 (80%)
Not available	5 (10%)
**BRCA status**	
BRCA 1 mut	5 (10%)
BRCA 2 mut	2 (4%)
VUS	3 (6%)
Negative	5 (10%)
Unknown	35 (70%)
**Type of surgery at diagnosis**	
Upfront	37 (74%)
IDS	11 (22%)
No surgery (exploratory laparotomy)	2 (4%)
**Postoperative tumor residual at first surgery**	
CC-0	16 (32%)
CC > 0	25 (50%)
Unknown	9 (18%)
**Median PFS at primary recurrence (months, range)**	7.5 (0–33)
**Primary platinum-resistant**	
Yes	20 (40%)
No	30 (60%)
**Treatment at first recurrence**	
Not surgery	32 (64%)
Surgery	18 (36%)
**CT before surgery for platinum resistance**	
Yes	32 (64%)
No	18 (36%)
**Median PFI from last platinum regimen (months, range)** (information available for 31 cases, missing in 19)	3.0 (0–5.0)
**Median number of previous CT lines before surgery for platinum resistance (*N*, range)** (information available for 49 cases, missing in 1)	3 (1–7)
**Median number of previous surgeries before surgery for platinum resistance (*N*, range)**	1 (1–4)

Legend: *N* = number; FIGO = International Federation of Gynecology and Obstetrics; BRCA = breast cancer; VUS = variant of unknown significance; IDS = interval debulking surgery; LPT = laparotomy; CC = completeness of cytoreduction; PFS = progression-free survival; CT = chemotherapy; PFI = platinum-free interval at first recurrence.

**Table 2:** Patient characteristics at platinum resistance and surgical procedures.

Variables	Patients *N* = 50 (%)
**Median age at surgery for platinum resistance (years, range)**	56.5 (35.6–78.4)
**Isolated relapse**	
No	48 (96%)
Yes	2 (4%)
**Relapse at platinum resistance ^a^**	
Peritoneum	31 (62%)
Lymph node	22 (44%)
Pelvis	8 (16%)
Liver	8 (16%)
Bowel	7 (14%)
Omentum	4 (8%)
Spleen	3 (6%)
Sigma rectum	3 (6%)
Diaphragm	2 (4%)
Paraombelical lesion	2 (5%)
Vagina	1 (2%)
**Intraoperative ascites at surgery for platinum resistance**	
No	27 (54%)
Yes	17 (34%)
Unknown	6 (12%)
**PCI at surgery for platinum resistance**	
<16	23 (46%)
≥16	20 (40%)
Not available	7 (14%)
**Upper abdomen procedures during surgery for platinum resistance**	
Yes	21 (42%)
No	28 (56%)
Unknown	1 (2%)
**Post-operative tumour residue at surgery for platinum resistance**	
CC-0	27 (54%)
CC > 0	21 (42%)
Unknown	2 (4%)
**Median duration of surgery for platinum resistance (minutes, range)** (information available for 39, missing in 11)	170 (30–660)
**Major morbidities of surgery for platinum resistance**	
Pneumothorax	5 (10%)
Acute respiratory failure	2 (4%)
Lymphocele	2 (4%)
Parenteral nutrition	2 (4%)
Renal insufficiency and electrolytic imbalance	2 (4%)
Infection/sepsis	2 (4%)
Pleural effusion	1 (2%)
Gastric laceration	1 (2%)
Subocclusion	1 (2%)
Pielostomy	1 (2%)
**30-day mortality**	4 (8%)
**Surgical procedures performed ^b^**	
Intestinal resection	22 (44%)
Peritonectomy	18 (36%)
Omentectomy	16 (32%)
Pelvic LND	12 (24%)
Paraortic LND	12 (24%)
Splenectomy	8 (16%)
Definitive stoma	7 (14%)
Liver resection	5 (10%)
Cholecystectomy	5 (10%)
**Post-operative CT**	
Yes	20 (40%)
No	20 (40%)
Unknown	10 (20%)
**Other treatments**	
Yes	14 (28%)
No	26 (52%)
Unknown	10 (20%)
**OS (months)**	
Median, 95% CI	23.0 (14.1–31.8)

Legend: *N* = number; PCI = peritoneal carcinomatosis index; CC = completeness of cytoreduction; LND = lymph node dissection; CT = chemotherapy; OS = overall survival from surgery for platinum resistance, CI = confidence interval. ^a^: several relapses are possible in the same patient. ^b^: several procedures are possible in the same patient.

**Table 3:** Multivariate analysis for OS.

Heading Title	*p* Value	HR	95% CI
**CC > 0 vs. not**	0.016	11.1	1.6–78.5
**Post-operative CT vs. not**	0.9	0.9	0.2–5.0
**Intra-operative ascites vs. not**	0.65	0.65	0.1–4.0
**PCI > 16 vs. not**	0.8	1.3	0.2–9.3
**Upper abdomen procedures**	0.5	0.5	0.1–2.9
**Age > 65 years vs. not**	0.2	2.5	0.6–10.0
G3 *	NA	NA	NA
**FIGO stage ≥ IIIC**	0.2	0.2	0.02–1.9

* Multivariate analysis not possible as G3 resulted in a dependent variable. Legend: CI = confidence interval; CC = completeness of cytoreduction; CT = chemotherapy; PCI = peritoneal carcinomatosis index; FIGO = International Federation of Gynecology and Obstetrics; G = grading.
